# An indoor air filtration study in homes of elderly: cardiovascular and respiratory effects of exposure to particulate matter

**DOI:** 10.1186/1476-069X-12-116

**Published:** 2013-12-28

**Authors:** Dorina Gabriela Karottki, Michal Spilak, Marie Frederiksen, Lars Gunnarsen, Elvira Vaclavik Brauner, Barbara Kolarik, Zorana Jovanovic Andersen, Torben Sigsgaard, Lars Barregard, Bo Strandberg, Gerd Sallsten, Peter Møller, Steffen Loft

**Affiliations:** 1 Section of Environmental Health, Department of Public Health, Faculty of Health and Medical Sciences, University of Copenhagen, Farimagsgade 5A K, DK-1014 Copenhagen, Denmark; 2 Danish Building Research Institute, Department of Construction and Health, Aalborg University, Copenhagen, Denmark; 3 Department of Environmental and Occupational Medicine, University of Aarhus, Aarhus, Denmark; 4 Department of Occupational and Environmental Medicine, Sahlgrenska University Hospital and Academy, Gothenburg, Sweden

**Keywords:** Endothelial function, Indoor air pollution, Intervention, Inflammation, Lung function, Particles

## Abstract

**Background:**

Exposure to particulate air pollution increases respiratory and cardiovascular morbidity and mortality, especially in elderly, possibly through inflammation and vascular dysfunction.

**Methods:**

We examined potential beneficial effects of indoor air filtration in the homes of elderly, including people taking vasoactive drugs.

Forty-eight nonsmoking subjects (51 to 81 years) in 27 homes were included in this randomized, double-blind, crossover intervention study with consecutive two-week periods with or without the inclusion of a high-efficiency particle air filter in re-circulating custom built units in their living room and bedroom. We measured blood pressure, microvascular and lung function and collected blood samples for hematological, inflammation, monocyte surface and lung cell damage markers before and at day 2, 7 and 14 during each exposure scenario.

**Results:**

The particle filters reduced the median concentration of PM_2.5_ from approximately 8 to 4 μg/m^3^ and the particle number concentration from 7669 to 5352 particles/cm^3^. No statistically significant effects of filtration as category were observed on microvascular and lung function or the biomarkers of systemic inflammation among all subjects, or in the subgroups taking (n = 11) or not taking vasoactive drugs (n = 37). However, the filtration efficacy was variable and microvascular function was within 2 days significantly increased with the actual PM_2.5_ decrease in the bedroom, especially among 25 subjects not taking any drugs.

**Conclusion:**

Substantial exposure contrasts in the bedroom and no confounding by drugs appear required for improved microvascular function by air filtration, whereas no other beneficial effect was found in this elderly population.

## Background

Exposure to particulate air pollution increases mortality and morbidity related to respiratory and cardiovascular diseases especially among susceptible individuals such as the elderly and people with pre-existing lung- and heart disease [1]. The underlying biological mechanisms are considered to include oxidative stress and inflammation. For cardiovascular disease these mechanisms include the pulmonary release of inflammatory and vasoactive molecules into the circulation, altered cardiac autonomic function, altered balance between coagulation and fibrinolysis, endothelial and microvascular dysfunction, atherosclerosis progression and plaque instability [2]. Ultrafine particles (diameter less than 100 nm), especially from combustion processes in vehicles, are thought to have particularly detrimental effects due to high alveolar deposition, poor clearance, large reactive surface area with attached metals and polycyclic aromatic hydrocarbons (PAH) on their carbonaceous core, and potential for translocation to the systemic circulation [3].

Health effects of exposure to air pollution is mainly related to outdoor levels, monitored or modeled in areas around residences. However, most people, especially those from the susceptible groups spend over 80–90% of their day indoors [4,5]. Although traffic-related particles are transported into the indoor environment by ventilation and infiltration [6], this is highly variable and a large proportion of indoor particles comes from a variety of indoor sources (frying, candles burning, heating devices, environmental tobacco smoke, office equipment, chemical reactions, biological sources and human activity), which may account for the majority of total personal exposure [7-9]. Biomarkers related to risk of cardiovascular and other diseases appear more strongly associated with personal exposure to particles than with ambient particulate air pollution levels [10,11]. Yet, little is known about the adverse effects of exposure to indoor-generated particles, except for the indoor allergens and aerosols on individuals with respiratory allergies or asthma [12]. Substantial reductions in exposure to particles can be achieved by portable air filtration units in the indoor environment allowing assessment of causality, understanding mechanistic endpoints, identification of relevant sources and development of large scale long-term interventions in relevant risk groups.

In a study of elderly (60–75 years) people, an 8% improvement in microvascular function measured by EndoPAT2000 (MVF) was detected following 48 hours of air filtration in their homes [13]. Similarly, significant improvement of MVF and a reduction in biomarkers of inflammation were found in 45 subjects aged 20 to 63 years after one week indoor air filtration in homes in wood smoke-impacted areas [14]. A recent review concluded that residential air filtration can improve outcomes in the treatment of allergic respiratory diseases [12]. Lung function was improved in young adults after filtration of tobacco smoke-polluted indoor air in the home, whereas the MVF was unchanged [15].

The objective of this study was to test the hypothesis that prolonged intervention with indoor air filtration of PM in the home improves MVF and lung function in an elderly representative non-smoking population, including people taking vasoactive and other drugs. In two consecutive two-week periods re-circulating custom built particle filtration units with the inclusion of a high-efficiency particle air (HEPA) alternating with sham filters were installed in the living room and bedroom. Secondary endpoints assessed included blood pressure, markers of systemic inflammation in terms of C-reactive protein and white blood cell counts previously shown to be affected by indoor air filtration [13,14], plasma levels of Clara cell pneumoproteins (CC16) and surfactant protein D (SPD) as sensitive biomarkers of epithelial damage in the lower airways [16,17], and the expression of surface adhesion molecules in terms of CD11b, CD31, CD49 and CD62L on circulating monocytes by flow cytometry, because monocyte activation with adherence to the endothelium is an important event in the atherosclerotic process [18].

## Materials and methods

The study protocol was approved by the regional ethics committee for human studies in Copenhagen (H-4-2010-102). All participants gave written informed consent prior to enrolment in the study.

### Study subjects and design

We recruited 51 (22 couples and 7 singles) non-smoking volunteers aged over 51 years and living in Greater Copenhagen in non-smoking apartments within 350 m (min 25 m, max 1000 m) from major roads (>10,000 vehicles per day) using radio spots as well as notices in local newspapers, activity centers and supermarkets. One couple and a single person later resigned, leaving 21 couples and 6 singles (22 men and 26 women) aged 51 to 81 years as the study population (Table 1). Eleven participants were taking vasoactive drugs (angiotensin-converting enzyme (ACE) inhibitors, calcium channel blockers, or β-adrenoreceptor blockers), 11 participants were taking statins and 12 participants were taking cyclooxygenase inhibitors (acetyl salicylic acid, ibuprofen or paracetamol). Two participants had diagnosed asthma and one participant had diabetes treated with metformin. According to diaries the participants spend in median (5^th^, 95^th^ percentiles) 83% (67%, 92%) of their time at home. This was similar for the periods with and without filtration 83% (63%, 92%) and 83% (67%, 92%), respectively.

**Table 1:** Characteristics of the study participants

**Characteristics of participants**	**Men**	**Women**	**Total**
Gender	22	26	48
Age (years)	67.7 ± 6.6	66.4 ± 6.6	67 ± 6.5
Height (cm)	180 ± 6.2	167 ± 5.0	173 ± 8.7
Weight (kg)	83 ± 12.2	69 ± 10.9	75 ± 13.4
Body mass index (kg/m^2^_)_	25.5 ± 3.1	24.6 ± 3.1	25 ± 3.1
Diastolic blood pressure (mmHg)	78 ± 7.7	77 ± 6.5	77 ± 7
Systolic blood pressure (mmHg)	127 ± 15.1	121 ± 11.8	124 ± 13.6
Total cholesterol (mmol/L)	3.7 ± 0.8	4.3 ± 1.1	4 ± 1.0
LDL cholesterol (mmol/L)	2 ± 0.5	2.2 ± 0.7	2.1 ± 0.6
HDL cholesterol (mmol/L)	1.1 ± 0.3	1.5 ± 0.4	1.3 ± 0.4
Triglycerides (mmol/L)	1 ± 0.5	0.8 ± 0.3	0.9 ± 0.4
Subjects taking vasoactive medication	9	2	11
Subjects taking statins	5	6	11
Subjects taking cyclooxygenase inhibitors	5	7	12
Subjects taking any drugs	12	11	23

Values are number or mean ± SD or numbers. LDL, Low-density lipoprotein; HDL, High-density lipoprotein.

The project was designed as a double-blind cross-over intervention with randomized order of exposure to re-circulated particle-filtered or sham-filtered indoor air in the home of the participants. Each exposure lasted for a 14-day period and the subjects served as their own controls, excluding confounding by characteristics that are stable within an individual over time, but vary between participants. All measurements were completed within a 7-month period starting November 2010, which corresponds to the same seasonal period as in our previous similar study carried out 4 years earlier [13].

Indoor air was re-circulated within each exposure period using custom built units with or without the inclusion of a HEPA filter class H11 (EN1822) placed in the living room and bedroom of each home. In the period with sham filtration, we used a dummy filter that conferred the same pressure drop and noise level. The participants as well as the researcher measuring health outcomes were blinded to the exposure scenario. Information about health, lifestyle housing and indoor climate were registered using self-administered questionnaires.

We measured MVF and lung function and all biomarkers in the morning at baseline (day 0) before start of air recirculation, and day 2, 7 and 14 of each exposure scenario. The same experienced researcher determined MVF and lung function and collected blood samples in all the participants’ homes.

### Exposure assessment

Particle number concentrations (PNC) were continuously monitored with a time resolution of 16 seconds with Philips NanoTracer1000 (Philips Aerasense, Eindhoven, Netherlands). This instrument detects PNC and average diameter of particles between 10 and 300 nm according to the manufacturer. In each home one instrument was placed in the living room for 24 hours at baseline, before turning on the air re-circulating units and in the end of each exposure scenario.

The level of PM_2.5_ (mass of particulate matter with aerodynamic diameter less than 2.5 μm) was measured in the bedroom and living room of each home. BGI 400 pumps collected air samples continuously at a constant flow rate (4 L min^-1^) through cyclone sampling heads GK 2.05-KTL (BGI Incorporated, USA). Suspended matter in the PM_2.5_ range was separated on Zefluor W/PAD 37 mm filter membranes (Sigma-Aldrich) replaced weekly. The PM_2.5_ mass was determined gravimetrically and the mass data have recently been reported elsewhere [19]. The material on the filters from the second week of each exposure scenario was also analyzed for black carbon (BC) at 880 nm and ultraviolet-absorbing particulate matter (UVPM) at 370 nm thought to be a measure indicative of aromatic organic compounds by Dual-Wavelength Optical Transmissometer using a Magee Aethalometer AE22 (© Magee Scientific Corp., Berkeley, CA) and PAH content using gas chromatography–mass spectrometry (GC-MS) [20]. PAH were sampled on filter only, therefore the levels of more volatile PAH (2–4 rings) may have been underestimated due to losses during sampling.

### Measurement of microvascular and lung function

MVF was measured non-invasively by peripheral arterial tonometry using the portable EndoPAT2000 (Itamar Medical Ltd, Cesaria, Israel), as previously described [21]. The method quantifies vascular function changes in the digital pulse waveform (PAT signal), measured with a pair of bio-sensors placed on the index finger of each hand. With participants seated in a quiet room a blood pressure cuff was placed above the elbow on one arm, while the contra-lateral arm served as a control arm. Resting blood pressure was measured using a WelchAllyn DuraShock DS54 manometer (Welch Allyn GmbH & Co. KG, Deutschland) before each MVF measurement. The EndoPAT2000 measurement consisted of a five minutes baseline, five minutes ischemic stimulus induced by supra-systolic cuff occlusion of flow through the brachial artery on the test arm and reactive hyperemia recorded for further five minutes after cuff deflation. The response to reactive hyperemia was calculated automatically through a computer algorithm as the ratio of the post and pre occlusion values normalized to measurements from the control arm.

Spirometry was performed in accordance with the American Thoracic Society/European Respiratory Society standard guidelines [22] using the NDD EasyOne Plus spirometer (ndd Medical Technologies; Zurich Switzerland). Forced expiratory volume in first second (FEV_1_) and forced vital capacity (FVC) were collected after MVF measurements. The data were digitally stored and the largest FVC and FEV_1_ from at least three acceptable maneuvers were used; the ratio of FEV_1_ to FVC was calculated.

### Biomarkers in blood

Blood samples were collected on the day of home visits. Peripheral venous blood samples were collected in CPT™ tubes with sodium heparin (BD Vacutainer® CPT™, Becton Dickinson A/S, Brøndby, Denmark) for peripheral blood mononuclear cells (PBMC) isolation and in EDTA tubes for hematological analyses. Within four hours we measured hemoglobin and leukocyte counts and their differential profile: lymphocytes, monocytes, granulocytes with a multiplatform analyzer (Chempaq XBC, by Denmark). We separated PBMC for storage at −80°C in freezing media consisting of 50% fetal bovine serum (FBS, GibcoRBL), 40% culture medium (RPMI 1640, GibcoRBL) and 10% dimetyl sulfoxide for flow cytometry analyses.

Plasma C-reactive protein (CRP), total cholesterol, high-density lipoprotein, low-density lipoprotein and triglycerides were analyzed at the Department of Clinical Biochemistry, Copenhagen University Hospital.

The concentrations of CC16 and SPD in plasma were analyzed by ELISA (Human Clara Cell Protein ELISA kit from BioVendor Laboratory Medicine, Inc., Brno, Czech Republic) at the Department of Occupational and Environmental Medicine, Sahlgrenska University Hospital and Sahlgrenska Academy, University of Gothenburg.

Direct immunofluorescence of PBMCs was performed on a BD Accuri™ C6 flow cytometer with BD Accuri CFlow®Plus software (BD Bioscience, Brøndby, Denmark) with all 7 samples from each participant analyzed in the same batch. Specific surface staining of the activation status of monocytes was performed with fluorescein isothiocyanate (FITC)-conjugated anti-CD49d (ITGA4), + Allophycocyanin (APC)-conjugated anti-CD11b (Mac1α) and FITC-conjugated anti-CD31 (PECAM-1) + Phycoerythrin (PE)-conjugated anti-CD62L (L-selectin) mouse monoclonal antibodies (BD Bioscience, Brøndby, Denmark). BD Compbeads stained with individual fluorescent probes were used to set up compensation; unstained cells were used to determine the baseline fluorescence of the monocyte population and cells stained with isotype-matched FITC, PE and APC-conjugated human immunoglobulin G were used as negative controls.

The samples were quickly thawed at 37°C for 1–2 min, 10 ml warm cRPMI was added and the tubes were centrifuged at 400 g for 7 minutes. Cells were then resuspended in 1 ml warm cRPMI, counted with a hemocytometer and scored for viability by trypan blue exclusion. PBMCs were placed in round-bottom 96-well plates (ca. 10^5^ per well), stained for 30 min at 4°C, washed twice with stain buffer with centrifugation at 250 g for 5 min, resuspended in 100 μl stain buffer and analyzed immediately. Monocytes were selectively gated based on their characteristic forward scatter and side scatter properties. The expression of CD11b, CD31, CD62L and CD49d on monocytes was quantified as percentage of positive cells from each sample.

### Statistical analysis

We used linear mixed models (*xtmixed* procedure in STATA 12.0) to estimate the effect of air filtration presented as a percent change with 95% confidence intervals on log-transformed outcomes, accounting for correlation between repeated measurements within individuals and for correlation between individuals (couples) living at same residence. Separate models of the effect of filtration as categorical variable were fitted for each of the three time points during the two weeks with or without filtration and overall for each outcome, adjusted for baseline level, BMI, age, and gender because participants with incomplete data were included. The potential modifying effect of vasoactive medication, statins and cyclooxygenase inhibitors on the effect of filtration was tested by inclusion of an interaction term in the model and tested by Wald test. In addition, we modeled each outcome on the actual decrease in PM_2.5_ concentration measured in the bedroom and living room during the filtration period for each of the three time points and overall, adjusted for baseline level, BMI, age, and gender. All models were also run without inclusion of baseline values as sensitivity analyses. The study was powered to detect an 8% change in microvascular function among 37 participants with type I and II error levels of 5% and 10%, respectively.

## Results

A total of 48 people from 27 dwellings participated in the study. Forty-five participants had seven home visits and three participants only had five home visits, resulting in a total of 187 home visits with 330 sets of measurements. One baseline MVF test and 14 spirometer tests were not recorded due to instrument failure.

The air filtration intervention in the 27 study homes resulted in a reduction of 30% in PNC in the living room and close to 50% of PM_2.5_ in both living room and bedroom (Table 2). The efficacy of the filtration was variable with changes in PM_2.5_ ranging from a reduction of 24 μg/m^3^ to an increase of 7 μg/m^3^ in the bedroom and from a 20 μg/m^3^ decrease to a 4 μg/m^3^ increase in the living room, respectively. The reductions in PAH, BC and UVPM were similar to the PM_2.5_ reduction. The outcomes at baseline and day 2, 7 and 14 of the repeated measurements within each exposure scenario are presented in Table 3. The baseline MVF and FEV_1_/FVC ratio were in keeping with a relatively healthy elderly population. There was no significant effect of the intervention as categorical variable on the MVF or lung function on any day of the days studied during the two-week period.

**Table 2:** Particle exposure in the homes of the participants during sham and active indoor air filtration

**Filtration**	**Living room**		**Bedroom**	
	**Sham**	**Active**	**Sham**	**Active**
Particle number count (#/cm^3^)	7669 (3435–45866)	5352 (1241–56654)	ND	ND
PM_2.5_ total mass^a^ (μg/m^3^)	8.0 (3.4–20.7)	4.3 (0.2–12.2)	7.6 (1.4–19.1)	3.7 (0–14)
PM_2.5_ black carbon (BC) (ng/m^3^)	ND	ND	480 (199–1328)	219 (19.6–545)
PM_2.5_ UV absorbing PM (UVPM) (ng/m^3^)	ND	ND	361 (191–792)	201 (43.5–454)
PAH^b^ (ng/m^3^)	0.48 (0.06–2.4)	0.25 (0.02–2)		

Values are median (5^th^, 95^th^ percentile). ND: not determined.

^a^PM _2.5_ total mass data have recently been reported elsewhere [19].

^b^Sum of 13 different polycyclic aromatic hydrocarbons (PAH): phenanthrene, anthracene, fluoranthene, pyrene, benzo(a)anthracene, chrysene, benzo(b)fluoranthene, benzo(k)fluoranthene, benzo(a)pyrene, perylene, indeno(1,2,3-c,d)pyrene, dibenzo(a,h)anthracene, benzo(g,h,i)perylene.

**Table 3:** **Median (5**^**th**^**, 95**^**th **^**percentile) of the outcomes at baseline as well as according to each exposure scenario**

**Biomarkers**	**Baseline**	**Sham filtration**			**Active filtration**		
	**Day 0**	**Day 2**	**Day 7**	**Day 14**	**Day 2**	**Day 7**	**Day 14**
MVF	1.69 (1.07, 2.87)	1.74 (1.22, 2.60)	1.80 (1.30, 2.96)	1.72 (1.38, 2.56)	1.73 (1.20, 2.78)	1.69 (1.27, 2.63)	1.73 (1.32, 2.84)
Diastolic pressure, (mmHg)	80 (70, 90)	78 (60, 90)	80 (70, 90)	80 (60, 90)	80 (65, 90)	80 (60, 95)	77.5 (60, 90)
Systolic pressure, (mmHg)	130 (100, 150)	120 (90, 150)	120 (105, 140)	120 (100, 150)	125 (95, 140)	120 (95, 150)	120 (100, 140)
C-reactive protein (mg/L)	0.8 (0.2, 4.9)	0.9 (0.2, 3.6)	0.9 (0.2, 5.5)	0.9 (0.2, 4.5)	0.9 (0.1, 4.7)	1.0 (0.2, 5.1)	0.9 (0.2, 4.3)
Hemoglobin (mmol/L)	9.6 (7.8, 10.7)	9.3 (8.0, 11.2)	9.0 (7.9, 11.4)	9.1 (7.9, 10.8)	9.2 (8.0, 10.5)	9.1 (8.0, 10.3)	9.2 (7.6, 11.7)
Leukocytes (×10^9^cells/L)	5.6 (4.1, 8.3)	5.9 (4.1, 7.6)	5.6 (3.8, 8.3)	5.1 (3.8, 8.8)	5.7 (4.0, 8.1)	5.5 (3.9, 8.9)	5.8 (3.8, 7.8)
Lymphocytes (×10^9^cells/L)	1.9 (0.9, 3.5)	1.8 (0.9, 3.4)	2.0 (0.8, 3.5)	1.9 (1.0, 3.2)	2.1 (1.1, 3.0)	2.0 (1, 3.6)	1.9 (0.9, 3.0)
Monocytes (×10^9^cells/L)	0.6 (0.4, 0.9)	0.6 (0.4, 0.8)	0.6 (0.3, 0.9)	0.6 (0.4, 0.9)	0.6 (0.4, 0.9)	0.6 (0.4, 0.9)	0.6 (0.4, 0.9)
Granulocytes (×10^9^cells/L)	3.0 (2.0, 4.8)	3.2 (1.9, 4.9)	2.6 (1.8, 5.3)	2.9 (1.9, 5.5)	2.9 (1.7, 4.9)	2.7 (1.8, 5.4)	3.0 (1.7, 5.4)
CD31(%)	93.8 (84.6, 97.9)	92.9 (85.2, 98.0)	93.6 (85.2, 98.1)	91.5 (81.1, 96.9)	94.3 (84.0, 97.9)	93.1 (79.4, 97.9)	92.0 (78.1, 97.9)
CD62L(%)	60.0 (40.7, 86.0)	63.6 (42.7, 79.0)	62.9 (44.1, 76.8)	61.9 (39.3, 77.9)	62.4 (45.7, 75.3)	60.7 (43.5, 80.7)	62.4 (44.1, 81.4)
CD11b(%)	33.7 (4.9, 63.7)	39.6 (4.0, 69.9)	41.8 (6.2, 76.2)	37.8 (12.6, 65.8)	41.5 (8.3,70.0)	35.8 (10.0, 62.6)	37.6 (6.7, 73.5)
CD49d(%)	60.5 (30.0, 95.5)	72.7 (32.4, 94.8)	76.7 (33.8, 94.3)	74.9 (32.6, 95.1)	72.4 (45.3, 96.5)	73.3 (30.7, 96.5)	71.5 (39.2, 95)
FEV1/FVC	0.72 (0.42, 1.20)	0.66 (0.47, 1.17)	0.74 (0.38, 1.20)	0.72 (0.45, 1.37)	0.74 (0.38, 1.20)	0.73 (0.46, 1.43)	0.73 (0.43, 1.18)
CC16 (ng/ml)	4.2 (2.0, 8.8)	3.5 (2.0, 9.4)	3.6 (2.0, 9.3)	4.0 (2.0, 8.36)	3.6 (2.0, 8.6)	4.3 (2.0, 10.7)	3.9 (2.0, 10.2)
SPD (ng/mL)	100.6 (42.0, 285)	100.6 (46.1, 302.8)	99.0 (51.2, 279.2)	97.6 (46.2, 283.8)	103.1 (42.6, 306.1)	94.3 (43.3, 294.2)	98.7 (50.9, 338.7)

MVF, microvascular function; CD, cluster of differentiation; FEV1, Forced expiratory volume 1 s (L); FVC, Forced vital capacity (L); CC16, Clara cell pneumoprotein 16; SPD, surface protein D.

The estimated overall differences between active and sham filtration were −1.4% and 0.8% for all days analyzed together for MVF and lung function, respectively (Table 4). On the individual measurement day 2, 7 and 14 of the study periods there were only small changes in both upwards and downward directions. Similarly there was no significant effect of the intervention on the biomarkers except for a 4% decrease in CD62L expression on day 2 of active filtration in contrast to a non-significant increase of 6% in this marker on day 14. For most of the remaining biomarkers the estimated changes were within a few percent with rather narrow 95% CI except for CRP, and CD11b expression that showed more variation, however, without sign of a filtration-related pattern. Results in terms of effect of air filtration as categorical variable were not sensitive to adjustment for the baseline measurement, window opening, age, gender, or BMI with very similar effect estimates with or without adjustment (data not shown).

**Table 4:** Percent changes (95% confidence interval) in outcome levels, according to exposure scenario comparing active to sham filtration estimated by mixed-effects models adjusted for BMI, age and gender

**Biomarkers**	**Day 2**	**Day 7**	**Day 14**	**Overall (all days)**
MVF	−1.4 (−9.1, 7.0)	−5.2 (−12.5, 2.7)	2.8 (−5.6, 12.0)	−1.4 (−5.8, 3.2)
C-reactive protein	3.7 (−15.3, 26.9)	−11.3 (−29.1,10.9)	−3.6 (−24.9, 23.8)	−4.5 (−15.5, 7.9)
Hemoglobin	−2.2 (−5.7, 1.4)	−2.1 (−5.5, 1.3)	−5.2 (−14.7, 5.3)	−3.2 (−6.9, 0.7)
Leukocytes	−0.7 (−6.6, 5.6)	−0.7 (−6.0, 5.0)	2.4 (−3.1, 8.3)	0.2 (−3.1, 3.6)
Lymphocytes	3 (−3.7, 10.1)	−0.6 (−7.5, 6.9)	3.4 (−5.1, 12.6)	1.7 (−2.4, 6.1)
Monocytes	−0.4 (−5.8, 6.1)	−1.5 (−7.1, 4.3)	2.2 (−4.5, 9.4)	0.05 (−3.3, 3.5)
Granulocytes	−3.8 (−12.4, 5.6)	0.5 (−7.9, 9.7)	2.4 (−5.9, 11.6)	−0.4 (−5.3, 4.7)
CD31	−0.01 (−1.4, 1.4)	−3.7 (−9.1, 2.1)	1.3 (−1.7, 4.4)	−0.8 (−3.1, 1.5)
CD62L	−4.4*(−8.4, -0.2)	−2.9 (−10.7, 5.6)	6.1 (−0.3, 12.9)	−0.4 (−4.2, 3.7)
CD11b	10.4 (−9.1, 34.3)	−6.2 (−19.3, 9.0)	−11.4 (−26.2, 6.4)	−3.1 (−12.5, 7.4)
CD49d	1.3 (−11.8, 16.4)	−2.2 (−9.1, 5.3)	−2.9 (−11.5, 6.5)	−1.3 (−6.9, 4.7)
FEV_1_/FVC	0.16 (−12.1, 14.1)	5.1 (−5.4, 16.8)	−2.2 (−9.2, 5.4)	0.8 (−5.1, 7.1)
CC16	−0.4 (−10.1, 10.3)	−1 (−13.4, 13.3)	0.2 (−8.8, 10.2)	−0.3 (−6.4, 6.1)
SPD	−0.2 (−7.3, 7.4)	−2 (−8.1, 4.5)	5.1 (−1.7, 12.5)	0.9 (−2.8, 4.7)

MVF, microvascular function; CD, cluster of differentiation; FEV_1_, Forced expiratory volume 1 s (L); FVC, Forced vital capacity (L); CC16, Clara cell pneumoprotein 16; SPD, surface protein D. *p<0.05.

There was no sign of modification of the effect assessed as active versus sham filtration by intake of drugs on outcome variables or different responses in any subgroups (Table 5; data only shown for MVF).

**Table 5:** **Percent change (95% confidence interval) in microvascular function comparing active to sham filtration and corresponding to a 10 μg/m**^**3**^**decrease in PM**_**2.5 **_**in the bedroom, estimated by mixed-effects models adjusted for BMI, age and gender and stratified according to medication**

**Medication**	**Active versus sham filter**		**Per 10 μg/m**^**3 **^**decrease in PM**_**2.5 **_**in the bedroom**	
	**Medication**	**No medication**	**Medication**	**No medication**
Vasoactive (n = 11)	−3.7 (−13.4, 7.0)	−0.7 (−5.6, 4.5)	−2.3 (−14.7, 11.8)	7.7 (2.3, 13.3)*
Statins (n = 11)	−0.8 (−8.8, 8.0)	−1.6 (−6.8, 3.9)	1.5 (−9.0, 13.3)	7.7 (1.1, 14.6)*
Cyclooxygenase inhibitors (n = 12)	−5.0 (−11.0, 1.5)	−0.1 (−5.7, 5.8)	4.2 (−2.1, 10.9)	7.8 (0.5, 15.5)*
Any drug (n = 23)	−3.17 (−8.8, 2.8)	0.37 (−6.4, 7.7)	3.3 (−3.9, 11.9)	8.4 (2.0, 15.1)*

*P < 0.05.

**Figure 1:**
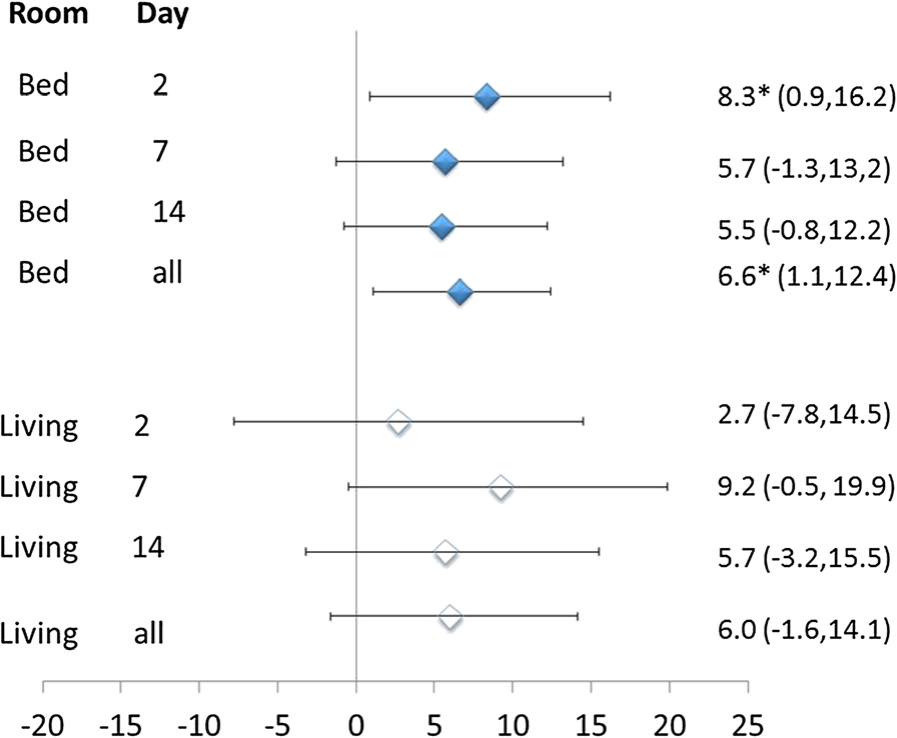
**Percent change with 95% confidence interval in microvascular function corresponding to a 10 μg/m**^**3 **^**decrease in PM**_**2.5 **_**in the bedroom and living room after 2, 7 and 14 days of active filtration and all 3 days combined, estimated by mixed-effects models adjusted for baseline level, BMI, age and gender.**

The variable efficacy in the filtration led to a post-hoc analysis of the effect of the actually achieved decrease in PM_2.5_ on the outcomes. This indicated that MVF was positively and significantly associated with the decrease in PM_2.5_ in the bedroom overall and on day 2 of the filtration period as well as in subjects not taking drugs, whereas there was no sign of such association among subjects taking vasoactive drugs (Figure 1 and Table 4). The decrease in PM_2.5_ in the living room showed a similar but less strong and not significant association with MVF (Figure 1). None of the other outcome variables showed significant associations with decreases in PM_2.5_, except CD62L, which showed a 9.4% (95% confidence interval: 1.5% to 18%) increase per 10 μg/m^3^ decrease in the living room (other data not shown).

## Discussion

We found unaltered MVF, lung function and biomarkers comparing active and sham re-circulating indoor filtration for two weeks in the homes of non-smoking elderly, including some with well-controlled vascular disease. With three measurements during each exposure scenario we are unlikely to have missed temporal changes. The indoor air filtration reduced the median PM_2.5_ concentrations from approximately 8 to 4 μg/m^3^, with similar decreases in BC and PAH that were already low, whereas the median PNC decreased from 7669 to 5352 particles/cm^3^. The filtration efficacy was variable with changes in PM_2.5_ ranging from a 7 μg/m^3^ increase to a 24 μg/m^3^ decrease. This led to a post-hoc analyses showing significantly improved MVF related to the decrease in the bedroom especially after two days filtration of the study period and in people not taking drugs, whereas the living room levels showed less strong and non-significant associations. The participants spent 83% of the time at home, which could not be restricted to the living room with air re-circulating unit during the daytime of the four weeks study. In comparison, our previous study with 48 hours air filtration reduced the PM_2.5_ and PNC from 13 to 5 μg/m^3^ and 10,000 to 3,200 particles/cm^3^, respectively. This was associated with improved MVF by 8% (95% CI: 0.4%, 16%) after two days in non-smoking elderly subjects, who did not take vasoactive drugs and stayed 92-94% of the time at home in only the two rooms with air filtration units [13]. Accordingly, our two air filtration studies on MVF among elderly appear compatible and even the 95% CI of the effects on MVF overlap related to active versus sham filtration. The difference in baseline levels of PM_2.5_ and PNC is partly consistent with decreasing outdoor PNC monitored at a busy urban street in Copenhagen in the period including our two filtration studies performed in 2006 (22,809 particles/cm^3^) and 2010/2011 (14,000 particles/cm^3^), respectively [23,24]. In the same period there has been increasing health consciousness because indoor smoking in public places has been banned (2007) and indoor sources of particles such as frying and candle use have been in public debate. Our realistic and relatively long air filtration intervention suggests that beneficial effects of further improvement of indoor air particle levels might be difficult to detect unless substantial exposure contrasts with possible emphasis on the bedroom are achieved. Nevertheless there was unaltered MVF among 37 young adults after filtration of indoor air with electrostatic filters in 20 homes polluted by tobacco smoke with a 37 μg/m^3^ reduction in PM_2.5_[15]. In contrast, significant improvement in MVF was found in 45 healthy subjects aged 20 to 63 years, after one week HEPA filtration of indoor air in the main activity room and the bedroom for seven days in non-smoking homes in wood smoke-impacted areas [14]. The filtration reduced PM_2.5_ from 11.2 to 4.6 μg/m^3^. Wood smoke is not likely to be particularly toxic because short-term exposures to 200–400 μg/m^3^ showed no effect on the MVF [25,26], whereas similar levels of diesel emissions have consistently shown impaired vasomotor responses [27,28].

Significant decreases in blood pressure and heart rate among young adults have been associated with 48-hours lasting reduction of indoor PM_2.5_ exposure after filtration reducing levels from around 24 to 18 μg m^3^ although not in a random study design [29]. Similarly, increased arterial blood pressure was associated with high ambient particle level and the heart rate variability and signs of ischemia were reduced by wearing a highly efficient facemask among patients with coronary heart disease [30]. However, we did not find effect of air filtration on blood pressure, which is consistent with our previous air filtration study and similar studies in wood smoke-impacted community and among First Nation Community [13-15].

High leukocyte counts and their subtypes can be biomarkers of vascular inflammation and predictor of coronary heart disease risk [31]. Short-term increases in ambient PM levels have been associated with elevated numbers of circulating leukocytes in the general population and patients with chronic pulmonary diseases [32,33]. In contrast, no effects on leukocytes or granulocytes were found after exposures to concentrated ambient air [34], diesel exhaust [35-37], or ultrafine particles [38]. In the present study, there was no effect of filtration on leukocyte count or their subtypes.

The air filtration was associated with decreased expression of CD62L on monocytes, although not consistently throughout the exposure period. This could well be a spurious finding because the CD62L expression was positively associated with the decrease in PM_2.5_ measured in the living room during the whole exposure period. The unaltered expression of adhesion markers CD11b, CD31 and CD49d on monocytes, suggests that pulmonary or systemic inflammation was not affected by the exposure contrast achieved in our study. This is in keeping with observations that 3-hours inhalation exposure to high concentrations of wood smoke particles had no apparent effect on surface marker molecules CD54 (ICAM-1), CD11a (ITGAL) and CD62L at 6- or 20-hours after cessation of the exposure [25]. However, 2-hours inhalation exposure to ultrafine carbon particles in healthy subjects was associated with reduced expression of adhesion molecules CD11b/CD18 on monocytes and CD11b/CD18 and CD49d on granulocytes [39]. Moreover, chronic biomass smoke exposure was associated with increased surface expression of CD11b/CD18 in circulating granulocytes and monocytes in Indian women [40]. The association between ambient air pollution exposure and expression of CD31 on monocytes has not previously been investigated in humans, whereas it has been shown that PM exposure in animals decreased CD31 and CD49d expression on circulating monocytes [41].

CRP is an important acute phase protein with pro-inflammatory properties associated with atherogenesis, modulation of endothelial function and risk of acute myocardial infarction [42] and it has been widely used as biomarker in air pollution studies [43]. We found no effect of filtration on CRP in keeping with our previous air filtration study in the home [13] and controlled short-term exposure studies of welding fumes, diesel exhaust and wood smoke [44-47]. In contrast, CRP decreased after filtration of the home air of residents in a wood smoke-impacted community [14]. Collectively, our observations from biomarkers of systemic inflammation including leukocyte counts, expression of adhesion markers on monocytes and CRP indicated minimal low-grade inflammation at baseline, which was not further reduced by indoor air filtration.

We found no effect of air filtration on lung function in the elderly population of the present study. Indeed, indoor air filtration appears mainly to alleviate airway symptoms in subjects with allergies and asthma [48], although electrostatic air filtration improved lung function in young adults from tobacco-polluted homes [15].

Plasma levels of CC16 and SPD are used as sensitive biomarkers of epithelial damage in lower airways [16,17]. We found no effect of air filtration on CC16 and SPD, suggesting intact epithelial barrier function at baseline and thus no effect of air filtration. Similarly, CC16 levels were not different after 24-hours exposure to polluted or filtered street air [49]. However, elevated CC16 levels have been observed after controlled exposure to wood smoke [50,51], whereas subjects using wood fuel for heating had lower levels of CC16 and increased SPD levels in a recent cross-sectional study [52].

We used a robust study design with 7-repeated measurements in a real-life setting. The limitations of this study include our limited statistical power and heterogeneous study population aged 51 to 81 years with some taking cardiovascular and/or cyclooxygenase inhibitor drugs. This might have attenuated potential effects, although stratified analyses showed no sign of differential effects comparing active and sham filtration and we had reasonable statistical power to detect relevant effects in subjects not taking any drugs. However, when accounting for variable filtration efficacy by using the actual decrease achieved in PM_2.5_ in the bedroom we found a positive effect especially among those people not taking vasoactive or to some extent other drugs, although there was no statistically significant interaction. Nevertheless, this suggests that effects of PM on vascular function might be masked by vascular drugs per se or more difficult to detect because of the existing disease they are taken for, similarly to the lack of effect of diesel exhaust exposure on an already poor vasomotor function among patients with ischemic heart disease [37]. It should also be considered that although this association between the PM_2.5_ decrease and improved MVF is consistent with our previous study, it was found in a post-hoc analysis and was not associated with changes in biomarkers that could explain possible mechanisms related to inflammation and monocyte activation. Genetically determined susceptibility can also modify cardiovascular effect of PM exposures [53,54]. Most importantly, the exposure contrast was small because the base-line particle concentration was already relatively low, albeit realistic exposures in current homes and the participant’s whereabouts were not restricted to the monitored rooms. Future studies of efficacy of air filtration could focus on homes with high levels of exposure due to outdoor and/or indoor sources and preferably selected by measurements in the home before the trial. Moreover, our findings related to the achieved decrease in PM_2.5_ suggest that the bedroom is the most important part of the home for the intervention and that effects on MVF can be expected within 48 hours of filtration. Unfortunately, personal monitoring of exposure in- and outside the home was not possible in our study, although individual time-activity patterns as well as the locations visited determine personal exposure to ambient air pollution [55]. Nevertheless, personal exposure to PM_2.5_ showed strong correlation with residential concentrations even when only 58% of the time was spent at home [56].

Furthermore, we measured MVF, in the homes of the participants, whereas forearm plethysmographic methods applicable in clinical settings might be more sensitive for detection of vasomotor dysfunction [28]. Finally, we measured a limited number of biomarkers of inflammation, monocyte activation and lung cell integrity at already low levels and effects on higher particle-induced levels or on other functions cannot be excluded [2,30].

## Conclusion

By comparing active and sham filtration of indoor air in the bedroom and living room for two weeks we found no improvement in MVF or lung function or detectable reduction in systemic inflammation, monocyte activation or lung cell damage in this elderly population, including people taking vasoactive medication, but with relatively low initial exposure levels. However, the filtration efficacy was variable and microvascular function was within 2 days significantly associated with the actual PM_2.5_ decrease achieved in the bedroom, particularly among subjects not taking any drugs, suggesting that positive effects of filtration require substantial exposure contrasts especially in the bedroom and possibly not confounding by drug intake or existing disease.

## Abbreviations

BC: Black smoke; BMI: Body mass index; CC16: Clara cell pneumoprotein 16; CD: Cluster of differentiation; CRP: C-reactive protein; FEV1: Forced expiratory volume in 1 second; FVC: Forced vital capacity; HEPA: High-efficiency particle air; MVF: Microvascular function; PAH: Polycyclic aromatic hydrocarbons; PBMC: Peripheral blood mononuclear cells; PM2.5: Particulate matter with aerodynamic diameter less than 2.5 μm; PNC: Particle number concentration; SPD: Surfactant protein D; UVPM: Ultraviolet-absorbing particulate matter.

## Competing interests

The authors declare that they have no competing interests.

## Authors’ contributions

SL, LG and TS contributed substantially to the concept and the design of the study. DGK was involved in the design and was responsible for ethical committee approval, recruitment of subjects, coordination, conduction of the study; acquisition of health-related data. MS, MF, BK and LG were responsible for the equipment for particle filtration, assessed indoor air quality and measured PNC and PM _2.5_ BS, GS and LB were responsible for the analysis of CC16, SPD, BC and PAH. DGK, PM and SL analysed and interpreted the health-related data with statistical support from EVB and ZJA. DGK drafted the manuscript, which was critically revised by SL and PM. All authors have read, corrected, and approved the manuscript.
